# “I AM THOR/DUST DAHO”: mnemonic devices used by the Paris Fire Brigade to teach initial measures in undertaking a CBRN event

**DOI:** 10.1186/s13054-021-03539-z

**Published:** 2021-03-22

**Authors:** Louise Giaume, Yann Daniel, Franck Calamai, Clément Derkenne, Romain Kedzierewicz, Aude Demeny, Kilian Bertho, Stéphane Travers, Bertrand Prunet, Fréderic Dorandeu

**Affiliations:** 1 Emergency Medical Department, Paris Fire Brigade, 1 Place Jules Renard, 75017 Paris, France; 2 1re chefferie du service de Santé, French Military Health service, Villacoublay, France; 3 Val-de-Grâce Military Health Academy, 1 Place Alphonse Laveran, 75005 Paris, France; 4 French Military Biomedical Research Institute, French Military Health service, 91220 Bretigny-sur-Orge, France

## Background

**Fig. 1:**
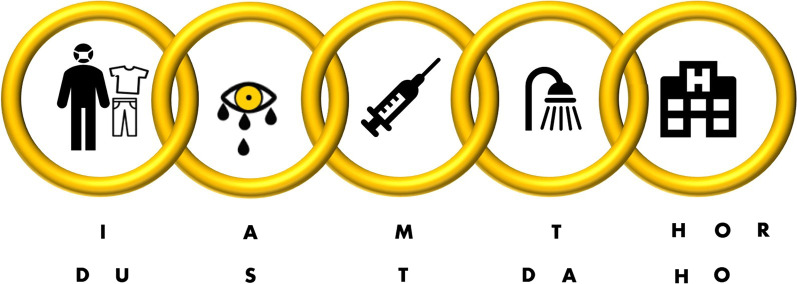
The CBRN chain of survival: the five essential tasks that first responders should perform in managing individuals in a CBRN situation

In 2019, the Paris Fire Brigade described the chemical, biological, radiological, and nuclear chain of survival (CBRN-CS) to point out five essential tasks that first responders should perform in managing individuals in a CBRN situation [1]. These tasks are inseparable, and they should be performed in the following order (Fig. 1): 1. Spot decontamination to reduce toxicity and prevent the spread of contamination; 2. Early toxidrome recognition to warn and prepare the healthcare chain; 3. Early antidote administration to prevent death; 4. Thorough decontamination to protect the healthcare system; and 5. Evacuation and transport to the hospital to continue the medical management of casualties. The purpose of developing the CBRN-CS was to provide a pragmatic, didactic tool that could be used during a CBRN crisis by all first responders, of any profession, and that would be applicable to all CBRN situations.

In CBRN events, we face multiple challenges. Despite the indisputable, constant threat of real CBRN situations, fortunately, they are rare. Thus, most first responders have never been exposed to real CBRN situations. Moreover, first responders often have insufficient levels of training and knowledge, and few have the opportunity to attend CBRN training refresher courses or simulation courses [2]. We predict that a CBRN crisis will cause mass disruption, especially for the healthcare system, more than mass destruction (except in the case of a nuclear device detonation). In these unusual situations, first responders will experience extreme stress, and they must face the dilemma of triage. The use of personal protective equipment can increase stress [3], and it alters communication between team members [4].

It is well known that stress affects cognitive performance [5, 6]. Simulation studies conducted to test how clinicians deal with stressful scenarios have shown that performance can be affected by stress [7, 8]. In stressful situations, decision-making is most effective when those in charge have simple tools to help them remember the main tasks that must be performed.

### “I AM THOR”: a mnemonic device

In January 2020, we initiated a survey to evaluate the CBRN-CS as a cognitive tool in training first responders in Paris. We asked all participants to place the CBRN-CS items in the correct order, immediately after the course, and 6 months later. Our results from the first 74 participants, which included exclusively medical practitioners and nurses, showed that 84.7% could place all the CBRN-CS items in the correct order immediately after the course. However, 6 months later, only 55.5% of respondents (only 45 participants out of the initial 74) could reconstitute the CBRN-CS, with items in the correct order. Despite the small size of this test population, we concluded that we needed to improve our strategy for teaching the CBRN-CS. We aimed to make the CBRN-CS more accessible and easier to remember, especially under stressful and uncommon situations, which are rarely practiced.

Cognitive aids, like checklists or emergency manuals, can support clinicians in optimizing their actions during critical events [9]. In emergency settings, optimal implementation leads to better team communication [10] and improves compliance to the standard of care [11]. Mnemonic teaching is a pedagogic strategy that improves memorization, and this strategy frequently employs mnemonic devices. A mnemonic device can recap checklists, which is a hardy strategy for optimal performance in harsh environments. For example, since the 1990s, the US military has used the “MARCH”' mnemonic to teach Tactical Combat Casualty Care (TCCC) [12]. In that mnemonic device, each letter corresponds to a term in the diagnosis and treatment protocol, in the correct order of relevance, for managing a war-injured individual [13]. A study conducted in the French military examined caregivers that were suddenly placed, without acclimation, in a high-altitude setting, where their pulse oximetry decreased. With this mnemonic, they could manage patients properly, without missing a significant intervention [14]. The dissemination of the TCCC approach, and its equivalents, among modern warfare personnel has improved the probability that individuals with war-related injuries will arrive alive to the hospital [15].

Thus, based on this approach, we developed a French mnemonic device to make the CBRN-CS easier to remember. This device is called “I AM THOR”, and its French translation is “DUST DAHO” (Table 1). It is currently taught in all CBRN training sessions to all first responders that might be involved in CBRN events. Henceforth, we will use it to complement the CBRN-CS cognitive aids. The next step will be to evaluate its efficiency among first responders.

**Table 1:** “I AM THOR/DUST DAHO”: mnemonic devices for the CBRN chain of survival

I AM THOR		French version: DUST DAHO	
I	Immediate decontamination	DU	Décontamination d’Urgence
A	Assessment	S	Symptômes
M	Medication	T	Traitement
T	Thorough decontamination	DA	Décontamination Approfondie
HO	HOspital	HO	HOpital
R	Re-evaluation		

## Conclusion

Our ability to recall learned information is often impaired, when acute stress, time pressure, and unfamiliarity are associated with the management of rare, high-acuity critical events, like CBRN situations. During the first few hours, whether they are on the scene or in front of the hospital, first responders must remember the appropriate response, share the same objectives, and coordinate their actions, regardless of their profession and the CBRN situation.

In CBRN events, appropriate, rapid actions are critical to curb disorganization and to optimize the management of injured individuals. Adding the mnemonic device “I AM THOR” to the CBRN-CS training could increase long-term memorization by first responders and help them initiate the main tasks required in any given CBRN situation.

## Data Availability

Not relevant.

## Abbreviations

CBRN: Chemical, biological, nuclear, and radiological
CBRN-CS: Chemical, biological, nuclear, and radiological chain of survival
TCCC: Tactical Combat Casualty Care
